# Dating apps as health allies? Examining the opportunities and challenges of dating apps as partners in public health

**DOI:** 10.1136/medhum-2024-012901

**Published:** 2024-05-15

**Authors:** Jaime Garcia-Iglesias, Brian Heaphy, Sharif Mowlabocus, Neta Yodovich, Maurice Nagington, Karissa Patton, Sophie Atherton, Andrea Ford

**Affiliations:** 1 Centre for Biomedicine, Self and Society, Usher Institute, The University of Edinburgh, Edinburgh, UK; 2 School of Social Sciences, The University of Manchester, Manchester, UK; 3 Communication & Media Studies, Fordham University, New York, New York, USA; 4 Nursing, Midwifery, and Social Work, University of Manchester, Manchester, UK

**Keywords:** social science, health policy, sexually transmitted diseases

## Abstract

In recent years, dating apps have become important allies in public health. In this paper, we explore the implications of partnering with dating apps for health promotion. We consider the opportunities and challenges inherent in these collaborations, paying special attention to privacy, trust, and user care in a digital environment.

Despite their potential as targeted health promotion tools, dating apps raise significant ethical concerns, including the commodification of user data and privacy breaches, which highlight the complexities of blending healthcare initiatives with for-profit digital platforms. Furthermore, the paper delves into issues of discrimination, harassment and unequal access within these apps, factors which can undermine public health efforts.

We develop a nuanced framework, emphasising the development of transparent data policies, the decoupling of content moderation from health initiatives and a commitment to combat discrimination. We underscore the importance of embedding app-based health initiatives within broader care pathways, ensuring comprehensive support beyond the digital domain. This essay offers vital insights for public health practitioners, app developers and policymakers navigating the intersection of digital innovation and healthcare.

## Introduction

In November 2023, Grindr, the most popular dating app for men who have sex with men (MSM), announced its partnership with MPOWER (in Ireland) and the Equality Movement (in the country of Georgia) to allow app users to order free, postal HIV testing kits through the app (Harrison-Quintana 2023). This is the latest example of a trend whereby dating apps are moving beyond offering matchmaking services to becoming significant players in public health, specifically in the sexual health arena. In the wake of the COVID-19 pandemic, dating apps have experienced an unprecedented surge in usage: as many as 30% of adults in the USA have used a dating app (Duguay, Dietzel, and Myles 2024), and a recent survey by eHarmony projects that by 2040 up to 70% of relationships could commence online (Holtzhausen *et al* 2020). In this evolving landscape, apps have been lauded as having the potential to serve as valuable tools in public health (Arnold 2023). However, the use of apps for these purposes can be problematic as it requires negotiating challenges to do with trust, risk and supporting users in digital settings.

Most dating apps are for-profit businesses, operating in an industry with vastly differing data compliance and confidentiality requirements to those used in medicine and public health. Thus, issues of privacy, content moderation and user care must be considered when collaborating with them. It is from this perspective that we develop a critically reflexive approach for analysing health partnerships with dating apps; one that considers the material, political and social conditions that underpin these collaborations. As dating apps increasingly become key partners in public health, particularly in the area of HIV and other sexually transmitted infections (STIs), it is imperative to question their effectiveness, and the ethical challenges and potential pitfalls that may arise from their use for this purpose.

## Dating apps: tools for public health?

The term ‘dating app’ commonly refers to smartphone-based, digital applications that allow users to create a profile (including images) and to view the profiles of other users. Users can search for others based on shared relational interests, preferences and/or location and can contact one another. Dating apps are said to have given rise to ‘new forms of intimacy and affective connection’ (Gibson 2021) that blur the online-offline divide and mediate diverse forms of social, intimate and sexual interactions. One of the most characteristic features of contemporary dating apps is their use of the geolocative capacity of smartphones (Duguay, Dietzel, and Myles 2024). According to a recent Pew survey in the USA, the dating apps Tinder, Match and Bumble are the are most widely used across all demographics, whereas the use of Grindr and Her is more prevalent among lesbian, gay and bisexual (LGB) adults. LGB adults are twice as likely as heterosexuals to report using dating apps (51% vs 28%) (Vogels and McClain 2023).

While some researchers have sought to explore the link between dating app use and poorer health outcomes (including increased STI diagnoses or lower mental health outcomes), evidence of a causal link remains inconclusive (Holtzhausen *et al* 2020). However, dating apps have also emerged as key partners in public health across a range of domains. Some researchers argue that dating apps have afforded the reconfiguration of sexual dynamics (Hollingshead, Dowsett, and Bourne 2020), presenting new opportunities for users to see, engage with and represent themselves as intimate actors (Brubaker, Ananny, and Crawford 2016). In a systematic review, Cao *et al* (2017) argue that dating apps have several benefits for public health, including the ability to narrowly target specific groups. They found that dating apps have been useful in increasing awareness around HIV and other STI testing. An example of successful collaboration is the Building Healthy Online Communities (BHOC) initiative, which is a consortium of public health organisations that works with dating app businesses to identify and advocate for apps to implement health-promotion features, such as sexual health-related profile options, testing reminders and, most recently, the ability to order HIV tests via the platform. BHOC emphasises that a key objective of these partnerships is to ‘promote strategies on dating apps that are sustainable and reach high numbers of users, and require no or little ongoing investment by public health programmes’ (Hecht *et al* 2022).

In the recent 2022–23 mpox outbreak, Grindr—the most popular dating app among MSM—became a partner in public health on the issue (Holloway 2022). In a survey of mpox awareness in the UK, while it was likely that the majority of respondents gained knowledge of the virus from mainstream and LGB targeted media or informal sources, 13% of respondents reported that dating apps were an important source of mpox information (Paparini *et al* 2023). In this respect, dating apps may be particularly useful in informing underserved groups, such as men who have sex with men (MSM) but who do not identify as gay or bisexual, while also going some way towards reducing stigma related to sexual public health. Grindr also partnered with the Pan American Health Organization to disseminate information on mpox to LGB+ communities in the Region of the Americas and tackle misinformation (Pan American Health Organization 2023). At other times, using dating apps for public health may be more ‘hand-made’. Linn County Public Health in Iowa created ‘official’ profiles in a variety of apps to perform syphilis partner notification (ie, notify partners of a person who has been diagnosed with syphilis), arguing that ‘the way we used to connect just isn’t working anymore. We need another tool in our toolbox […] one better aligned with the many ways people now meet their partners’ (Arnold 2023). Reporting on this, Harvard Public Health magazine comments how dating apps could ‘become a key component in STI prevention’ (Arnold 2023). In some of these examples, dating apps have been effective in disseminating information to underserved groups, and in resource-limited settings where alternative interventions may not be feasible. This fact should be understood in terms of the history of successful HIV/AIDS prevention work in the UK, the USA and elsewhere in the global north, which provides clear evidence that health intervention tailored to specific communitie are especially effective when they take place in spaces already used by the target population. When translated into the contemporary digital landscape, it makes sense that dating apps, which have proven popular with gay, bisexual and MSM, have become a site for local scale public health activity.

Beyond sexual health matters, dating apps have played roles in addressing broader public health concerns. In the context of COVID-19, partnerships between leading dating apps and governments have been established to boost vaccine uptake, with in-app rewards for those sharing their COVID-19 vaccinated status by, for example, adding “I’m vaccinated” tags to their profiles (Department of Health and and Social Care 2021). Collaborations can also extend beyond infectious diseases: in the UK, Tinder has partnered with the UK Government to tackle loneliness through sharing messages about coping and support with users, and partnered with the National Health Service (NHS) to raise awareness of organ donation (NHS Blood and Transplant 2015; Tinder 2023). These examples highlight how, much like social media on a broader scale (Garcia-Iglesias *et al* 2023), dating apps offer distinctive opportunities for public health initiatives. Overall, there is evidence that apps provide numerous benefits for public health, particularly in the field of sexual health in terms of increasing awareness around STIs, facilitating testing and providing links to healthcare agencies while allowing for the narrow targeting of audiences.

## The challenges of dating apps in public health

Dating apps, like many other digital platforms, have a questionable track record when it comes to issues of data privacy. In their study of gender politics in social media and dating apps, Bivens and Haimson (2016) argue that despite platforms aiming to present themselves as ‘neutral or open online spaces’ designed with users’ benefit in mind, ‘their primary goal involves generating, capturing and controlling user data’ for financial profit, giving them substantial power over users (2). The fact that user data have today become the principal commodity through which profit is realised by digital platforms (witness, for instance, the targeted advertising found on Facebook or Instagram), is a long-standing concern which takes on a new dimension when that data could include information such as the number of HIV testing kits ordered, the addresses to which they are posted to and so on.

Striking a careful balance between safeguarding users’ privacy and maximising profit is crucial, with dating apps often being described as a ‘security and privacy minefield’ (O’Flaherty 2022). Numerous reports detailing incidents of stalking, data leakage, blackmail and even torture have been published since Grindr went online in 2007 (Noto La Diega, Guido 2018). These raise concerns that are not incidental to dating apps but are inherent to their design and feasibility. In a study of privacy in gay dating platforms, Campbell (2005) argues that although many operate on a ‘freemium’ basis, these platforms require users to ‘exchange personal information—in essence, barter their privacy—for the privileges of membership’ (Campbell 2005, 672). This becomes especially problematic when those platforms become providers of health resources, as using dating apps for public health can require individuals to ‘pay’ for such service by forgoing their privacy. This fits with a general trend in ‘universal’ healthcare systems (such as the NHS in the UK) towards a ‘privatisation by stealth’ of healthcare. While the user might not be exchanging money for services, they are required to ‘give up’ something of value: their personal information. Whether in the contexts of a universal healthcare system, or a consumer-based one, this trend can be especially concerning when the services offered are specifically directed to groups that are marginalised socially, economically and culturally, for example, by gender, race, disability, geographical location and/or age.

Examples of problematic data policies on dating apps abound and complicate some of the benefits highlighted earlier. For example, in 2018, journalists reported that Grindr had been sharing data that could identify their users, including HIV status, date of testing, email and location, with third party companies (Ghorayshi and Ray 2018). These are data which users were required to input if they wished to activate testing reminders. More recently, Grindr was fined €6.5 million by the Norwegian Data Protection Agency for disclosing data to advertising third parties without valid consent (European Data Protection Board 2021). These data policies have important personal and security ramifications, such as identifying specific users or outing them in unsafe local contexts (Tau and Wells 2022), and internationally where the consequences could be imprisonment, torture or even death (eg, Iran, Saudi Arabia, Nigeria).

While economically marginalised groups can be excluded from full access to the affordances of dating apps, as these usually require payment for a membership fee, research has also shown how apps have become hot-beds of discrimination and exclusion. This undermines users’ trust in dating apps, and can limit the reach of public health initiatives that are developed with them. Lauckner *et al* (2019) in a study of the experiences in dating apps of rural sexual minority men in the USA found that online dating often involved experiences of ‘deception, bullying or discrimination, and harassment or coercion’ both subtle and open (Lauckner *et al* 2019, 299). Li and Chen (2021) describe how Australian-based Chinese queer women have found dating apps to be permeated with racism and prejudice—even more so during COVID-19. Most worryingly, Conner (2023) has argued that these are not peripheral to app use but rather, that ‘mobile dating apps are structured to promote stigmatising other users and promote a sexual hierarchy rooted in heteronormative gender roles’ that disproportionately impacts minorities (Conner 2023, 126). For example, while some apps allow users to filter potential partners by factors such as HIV status, most rely heavily on pictures that allow for ‘informal’ discrimination in terms of race, gender, body type, age and so on.

Furthermore, dating apps operate with opaque policies. For example, the Terms of Service policies for Grindr and Tinder explicitly state that the apps reserve the right to ‘terminate any user’s account, for any reason and without any notice or our being liable to you’ (Grindr 2023) or ‘terminate your account at any time without notice [and without] any refund for purchases’ (Tinder 2021). These policies can complicate efforts to leverage these platforms for public health purposes. In fact, the US Centre for Disease Control and Prevention acknowledges, in their own toolkit for using dating apps for partner notification (to notify partners of a person who has been diagnosed with an STI), that no apps except for one (Adam4Adam) ‘have officially sanctioned the use of their apps for [organisational] partner services’ and that many organisations attempting to use them in this way ‘have had their profiles blocked on occasion by the managers of the app’ (Centers for Disease Control and Prevention 2022).

This can cause particular difficulties when public health and criminal justice overlap in relation to sexual health. For example, in London, UK, the charity *Controlling Chemsex* uses profiles to promote and discuss health issues relating to chemsex (the sexualised use of drugs). However, the people accessing services this way may, ‘without notice’ be banned from apps for discussing drug use, thus precluding them from accessing the information or support. The opaque policies about who can use dating apps and for what purposes means that their use as public health tools is a complicated issue.

In summary, leveraging dating apps for public health requires a high degree of negotiation between the app and the potential public health partner, and is likely to be marred by substantial drawbacks to do with trust and risk in dealing with digital businesses whose primary concern is profit, and whose practices can encompass questionable privacy policies, and damaging (and often unchecked) dynamics of exclusion (economic positioning, racism or harassment and opaque moderation policies). These challenges underscore how the incorporation of these platforms into public health initiatives is not a simple or straightforward matter.

## Towards a critically reflexive approach

In charting a path towards new approaches to dating apps and public health, it is crucial to avoid simplistic narratives that either vilify these platforms as contributors to STIs or uncritically deploy them for public health. Instead, we should recognise the increasing importance and utility of these platforms, particularly in resource-limited settings, and when seeking to engage underserved communities. In these contexts, dating apps are likely to become ever more instrumental in disseminating crucial health information. While acknowledging the benefits of partnering with dating apps, we call for a critically reflexive approach to such partnerships that reflects on and scrutinises the inherent tensions raised by their for-profit nature and business models. The approach we advocate foregrounds the following:

1. Acknowledgement that while partnerships with dating app businesses entail negotiations, ethical practice should not be an area of compromise.
2. Clear prioritising of the benefits of partnerships for dating app users, not the dating app business or the public health service.
3. Critical reflection on the ethical issues that arise in partnering with dating apps in public health, with emphasis on developing a transparent and publicly accessible policy detailing how the platform partner will handle user data.
4. This policy should mirror data protection guidelines currently used in clinical settings, and must include information regarding the timely, secure deletion of data.
5. Such a policy should explicitly confirm the platform’s requirement not to share user data (in any form) with third parties (including government agencies), and that these data will also be excluded from any direct or indirect form of monetisation activity (eg, targeted advertising sales).
6. The policy should be subject to review and approval by an ethics panel made up of peer experts.
7. There should be a decoupling of the platform’s standard content moderation policies and practices from public health initiatives, to ensure that users who seek support for practices that might run counter to the apps moderation policies (eg, about drug use) are neither censored nor penalised for discussing such practices.
8. An explicit commitment on the part of the dating app to monitor and address racial, gender, disability discrimination and body shaming, to ensure that users who seek support for their health do not face harassment, hate speech or intimidation.
9. The publication on the app of links to support services beyond the apps, to ensure that users understand the ways in which the partnership is embedded within a broader care pathway.
10. Publication of a periodic review of ethical issues encountered, how they were managed and implications for the partnership, to be considered and approved by expert ethics panel.

In summary, the use of dating apps has grown exponentially since they were first available, generating new forms of intimacy that often involve sexual practices. This requires a rethinking of how public health engages with and reaches its target base. While partnering with dating apps in sexual public health has clear advantages, not least in connecting with underserved populations, it also has its challenges. Here, we have focused on key ethical challenges linked to privacy, personal data, discrimination and exclusion, and argued that these are issues of paramount importance. We have offered an approach through which ethical partnerships can be thought about and managed: one that is based on and supports critical reflexivity.

## Data Availability

There are no data in this work.
